# Supplementary material for: Conditional chemoconnectomics (cCCTomics) as a strategy for efficient and conditional targeting of chemical transmission
Source: eLife. 2024 Apr 30;12:RP91927. doi: 10.7554/eLife.91927 (PMC11060718; doi:10.7554/eLife.91927)
Supplement: Figure 3—source data 3. [file elife-91927-fig3-data3.zip › Figure 3 source data3 part 2.zip!/Figure 3-Source data 3-part2/1052-13-CRISPResso_Report_ejedac2.zip!/CRISPResso_on_1052_16/2a.Nucleotide_percentage_quilt.pdf]

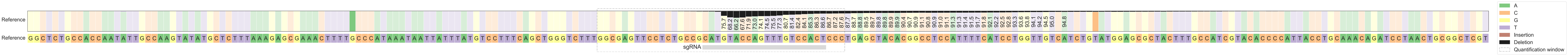
Reference
75.7 68.2 66.2 67.6 71.9 73.0 74.1 74.5 75.5 77.3 80.7 81.4 82.4 84.1 85.3 86.3 86.6 86.7 87.2 87.6 87.7 88.7 88.8 89.5 89.7 89.8 89.8 89.9 89.9 90.4 90.7 90.9 91.1 90.8 90.9 91.0 91.1 91.3 91.3 91.4 91.5 91.7 91.8 92.1 92.2 92.5 92.8 93.3 93.6 93.8 94.1 94.2 94.5 95.0 94.8
Reference
GGCTCTGCCACCAATATTGCCAAGTATATGCTCTTTAAAGAGCGAAACTTTTGCCCATAAATAATTATTTATGTCCTTTCAGCTGGGTCTTTGGCGAGTTCCTCTGCCGCATGTACCAGTTTGTCCACTCCCTGAGCTACACGGCCTCCATTTTCATCCTGGTTGTCATCTGTATGGAGCGCTACTTTGCCATCGTACACCCCATTACCTGCAAACAGATCCTAACTGCGGCTCGT
sgRNA
A
C
G
T
Insertion
Deletion
Quantification window
